# An appraisal of the principal concerns and controlling factors for Arsenic contamination in Chile

**DOI:** 10.1038/s41598-023-38437-7

**Published:** 2023-07-10

**Authors:** Mohammad Ayaz Alam, Abhijit Mukherjee, Prosun Bhattacharya, Jochen Bundschuh

**Affiliations:** 1 Departamento de Ingeniería Geoespacial y Ambiental, Facultad de Ingeniería, Universidad de Santiago de Chile, Enrique Kirberg Baltiansky n° 03, Estación Central, Santiago, Región Metropolitana Chile; 2 Department of Geology and Geophysics, Indian Institute of Technology Kharagpur, Kharagpur, West Bengal India; 3 KTH-International Groundwater Arsenic Research Group, Department of Sustainable Development, Environmental Science and Engineering, KTH Royal Institute of Technology, Stockholm, Sweden; 4 School of Engineering, Faculty of Health, Engineering and Sciences, University of Southern Queensland, West Street, Toowoomba, Queensland Australia

**Keywords:** Environmental sciences, Hydrology

## Abstract

Although geogenic Arsenic (As) contamination is well-recognized in northern Chile, it is not restricted to this part of the country, as the geological conditions favoring As release to the human environment exist across the country as well, although not at the same level, based on comparatively fewer studies in central and southern Chile. The present work provides a critical evaluation of As sources, pathways, and controls with reports and case studies from across the country based on an exhaustive bibliographic review of its reported geogenic sources and processes that affect its occurrence, systematization, and critical revision of this information. Arc magmatism and associated geothermal activities, identified as the primary As sources, are present across the Chilean Andes, except for the Pampean Flat Slab and Patagonian Volcanic Gap. Metal sulfide ore zones, extending from the country’s far north to the south-central part, are the second most important geogenic As source. While natural leaching of As-rich mineral deposits contaminates the water in contact, associated mining, and metallurgical activities result in additional As release into the human environment through mining waste and tailings. Moreover, crustal thickness has been suggested as a principal controlling factor for As release, whose southward decrease has been correlated with lower As values.

## Introduction

**Figure 1:**
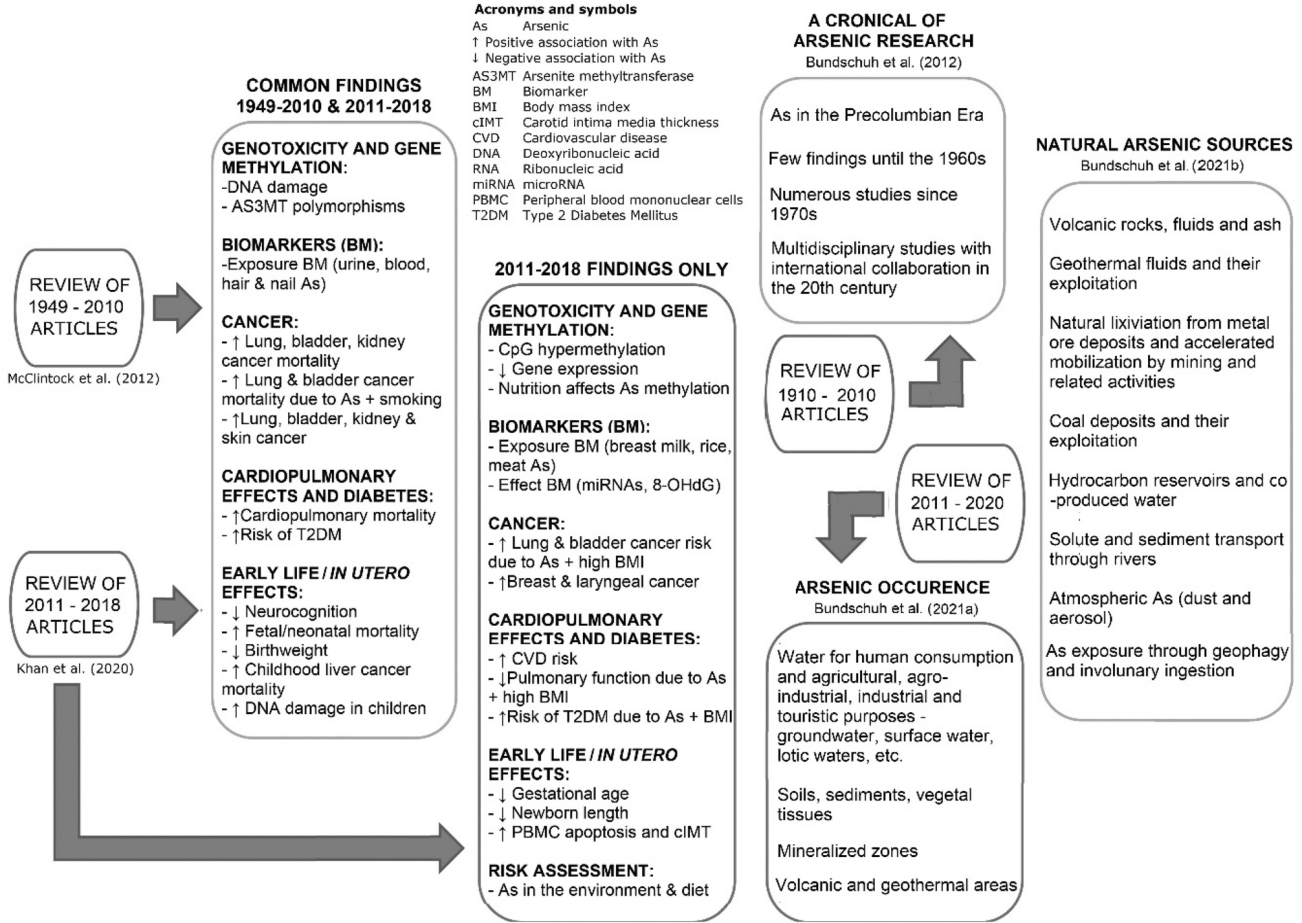
Findings of the two reviews of the studies on the health effects of As exposure in Latin America^1,2^, with many of the reported studies reported from the Antofagasta Region of Chile, together with three other reviews documenting evolution of As research in Latin America^4^, recent studies on its occurrence^3^ and natural sources^5^. Basic scheme from Fig. 2 of Khan et al.^1^ used with permission.

Most of the Latin American studies on the health effects of Arsenic (As) have been reported from Chile and Mexico^1^. However, in Chile, these studies have been limited to the Antofagasta Region. Two reviews^1,2^ of the articles on As health effects published between 1949 and 2018 in peer-reviewed journals show that the problem is aggravating even in this focused region despite more than half a century of efforts towards mitigation of human exposure to As (Fig. 1). These reviews documented the data on the biomarkers of As exposure, genetic susceptibility, genotoxicity, and risk assessment characterize the health effects and exposed populations. A synthesis of the findings of the studies from the Antofagasta Region is presented in "Arsenic-endemic Antofagasta Region".

Bundschuh et al.^3^ updated their earlier review^4^, with most of the coauthors of the previous work (see Fig. 1 for more details on the updated findings), of the history and occurrence of the As exposure in 14 Latin American countries known for over a century since the first confirmed modern case reported in Bell Ville locality in Argentina that got the name "Bell Ville Disease"^5^, renamed later as Endemic Regional Chronic Hydroarsenicism^6–8^. Bundschuh et al.^3^ further expanded the review to include the Caribbean and more recent studies on As sources, mobilization, and mobility in human environments affecting the region. Volcanic and geothermal activities, together with accelerated As release from geogenic sources in mineralized zones by mining and related activities, are the two most important and well-recognized sources and mechanisms for As release into environments affecting the Latin American population's environments^9^.

Within the distinct water types present in Chile, Tapia et al.^10–12^ reported the highest As concentrations in brines (88 mg/L) followed by saline (non-thermal, 29 mg/L) and thermal water (26 mg/L). They also observed this metalloid's natural presence in some volcanic rocks and sediments in the Arica and Parinacota, and Tarapacá administrative regions, including marine sediments. Moreover, elevated As concentration is reported in saline precipitates of the Atacama Desert (1420 mg/kg^13,14^), sinter deposits of El Tatio (21 wt%^15^) and Puchuldiza (60,189 mg/kg^16^) geothermal fields, evaporites of the Altiplano-Puna plateau^17^, high sulphidation epithermal deposits, viz. El Indio^18^, river sediments, viz. the Elqui River (55–485 ppm As^19–21^).

A study encompassing 15 Latin American countries by Morales-Simfors et al.^22^ reported As occurrence (0.001 < As < 73 mg/L, mean: 36.5 mg/L) in thermal waters from 423 geothermal fields, most of them associated with present-day volcanic activity. When mixed with the ground or surface water, these As-rich thermal waters contaminate these water resources^23^. An example of high As concentration (up to 50 mg/L^24,25^) in thermal waters in Chile is from El Tatio Geysers, which is the principal source of As in the Loa River^26,27^, a major surface water resource in the Atacama Desert, a continuous strip for nearly 1600 km along the narrow coast of the northern third of Chile, spanning over five administrative regions (Arica and Prinacota, Tarapacá, Antofagasta, Atacama, Coquimbo), from near Arica (18° 24′ S) southward to near La Serena (29° 55′ S)^28^.

Tapia et al.^29^ have studied the link between elevated As concentrations in the Altiplano-Puna region of Chile, consisting of its northernmost regions, with Mio-Pliocene to Quaternary volcanic activity, high crustal thicknesses, and geological structures. These authors hypothesized volcanism as an important As source and the thick continental crust a large As reservoir that can be leached by the thermal fluids upwelling through regional fault systems, leading to As concentration in the fractures. They further proposed that hydrological processes transport dissolved As to lower elevation regions through ground and surface water, where it is diluted in As in the absence of evapoconcentration prevalent in the highlands.

Moreover, Tapia et al.^10,11^ attributed decreasing values of As concentration towards southern Chile to dilution because of increased precipitation and the absence of As-rich evaporites, brines, and saline water present in northern Chile^19,30^. In another work, Tapia et al.^12^ attributed this trend to decreased shortening of the Andes in southern Chile, as related lower crustal thickness induces lower crustal contamination, acting as an As-poor provenance for the sediments and surface water. On the contrary, they reported a good correlation between crustal thickness and As concentrations in surface water and fluvial sediments in northern Chile, where crustal thickness in much higher (~ 75 km below the Altiplano region^31^) than that along the southern Andes (< 40 km^31^).

### Scope of the present work

Human exposure to As from geogenic and anthropic sources through water, sediments and soil is a significant health issue in Latin America and Chile (see "Arsenic situation in Chile"), and there is an urgent need to address this issue. The present work is an attempt towards delineation of the areas with anomalous As concentration in water and sediments across the country, based on the available geochemical dataset to decipher the processes and mechanisms of As-enrichment, as a part of a larger project “AGUA—Andean geoscientific network for strengthening environmental interdisciplinary research associated with water resources” (FOVI220217), funded by the National Agency for Research and Development (ANID) of Chile. Arsenic, detected at low concentrations in virtually all environments^32–36^, has been chosen to identify the naturally contaminated zones for being one of the most ubiquitous toxic elements present in the near-surface environment and exposed to humans through air, water, soil, and food chain^37^. On the other hand, groundwater As is selected in particular for the aforementioned project because of its high concentration present in a more toxic form, inorganic trivalent arsenite [As(III)]^38–40^.

Following decades of geological, geochemical, and epidemiological studies related to As contamination, northern Chile was identified as an As endemic; however, that does not imply that other parts of the country are uncontaminated. For example, Daniele et al.^41^ found bottled mineral water from cold springs in central and southern Chile contaminated, with up to 18.97 µg/L As and 3 of 10 popular brands having As concentration above the Chilean legal permissible value of 10 µg/L. This shows how supposedly pure waters are naturally contaminated, underscoring the need for a countrywide in-depth study to identify the As contaminated areas and decipher associated geological conditions. A recent study by Tapia et al.^12^ is a step forward in this regard.

### Materials and methods

The present review does not have a traditional structure, particularly to make it easier to read, considering it covers a wide range of topics on As situation in Chile. Nonetheless, we have followed the method of Mengist et al.^42^, which considers two additional steps besides four basic steps in the conventional scientific literature review method, viz. Search (i.e., defining searching string and types of databases), Appraisal (based on pre-defined literature inclusion and exclusion and quality assessment criteria), Synthesis (through data extraction and categorization), and Analysis (discuss the results and arrive at conclusions), known as SALSA^43^. The two additional steps in the modified method of Mengist et al.^42^ are defining a Protocol (defining the research scope, see "Scope of the present work") before applying SALSA^43^ method and it finishes with Reporting the results, as we have done by contextualizing the data with the geological setting. Accordingly, the modified method is known as PSALSAR^42^. We have used the PSLR method for the present appraisal of As situation in Chile, as it helps generate topic-specific existing knowledge, trends, and gaps that would be appropriate for policymakers and the scientific community, which is the purpose of the present work.

This search was conducted using a variety of databases, including Web of Science (WoS), Scopus, Science Direct, Springer, Google Scholar, PubMed, and Research Gate. These databases were searched for the "Topic" that includes "Title" and "Abstract" using the following combinations: "Arsenic" AND "Chile" AND "water", "Arsenic" AND "Chile" AND "soil", and "Arsenic" AND "Chile" AND "sediment". For example, WoS Core Collection provided 261, 70, and 49 results for the combinations above, respectively. Many of the results were common for the aforementioned search combinations. Articles published between 2000 and 2023 were considered in this search. Since water quality standards^40^, including As concentration limit, have changed through time worldwide, including in Chile, the journal articles, book chapters, or books published before this period were not included in this review. Since Chile has no legislation establishing legal limits on sediment and soil contamination, no specific criteria were used in this regard. Sporadic references to the works before 2000 are for complementary information regarding geology or were needed to complete the narrative. Following the collection of the published works, the titles and abstracts were scrutinized, and irrelevant ones were separated from the collection and kept in a separate folder to ensure the reproducibility of the work and reuse for future work. After that, full-text articles were analyzed to determine which were the most appropriate and relevant for the present work and stored in a database containing the articles cited here.

The following inclusion and exclusion criteria were used. Inclusion criteria: presence of As in water, soil, and/or sediment; As concentration reported; absolute, average value or range of As concentration presented; analytical method used for water as per WHO^40^ recommendations; prevalence data presented; publications between 2000 and 2023; papers written in English or Spanish (only SciELO journals in case of the latter). Exclusion criteria: any other metal contamination; only qualitative information; methodology nor presented; methodology for water not as per WHO^40^ recommendations; publication before 2010. Of the total 284 articles and reports, 69 were excluded for the aforementioned exclusion criteria or repetitive information.

Although we have used aforementioned peer reviewed scientific publications for the review work on As data in water, sediments and soil, some government decrees, parliamentary bills, resolutions, reports, or other documents by government agencies, reports by environmental organizations, and news reports in Spanish have been cited while referring to the government initiatives or the lack of it towards mitigation of environmental issues related As or other metal-metalloid contamination of water and soil, e.g., in the context of sacrifice or saturated zones (see "Sacrifice or saturated zones"). Such documents in Spanish have been referred directly by first author (MAA), who is multilingual (English, Spanish, and several other languages). Data on As in thermal waters has been cited to the original source, a report in Spanish^24^. Data from this report were used in a peer reviewed publication in 2011^25^.

## Arsenic situation in Chile

### Arsenic–endemic Antofagasta region

**Figure 2:**
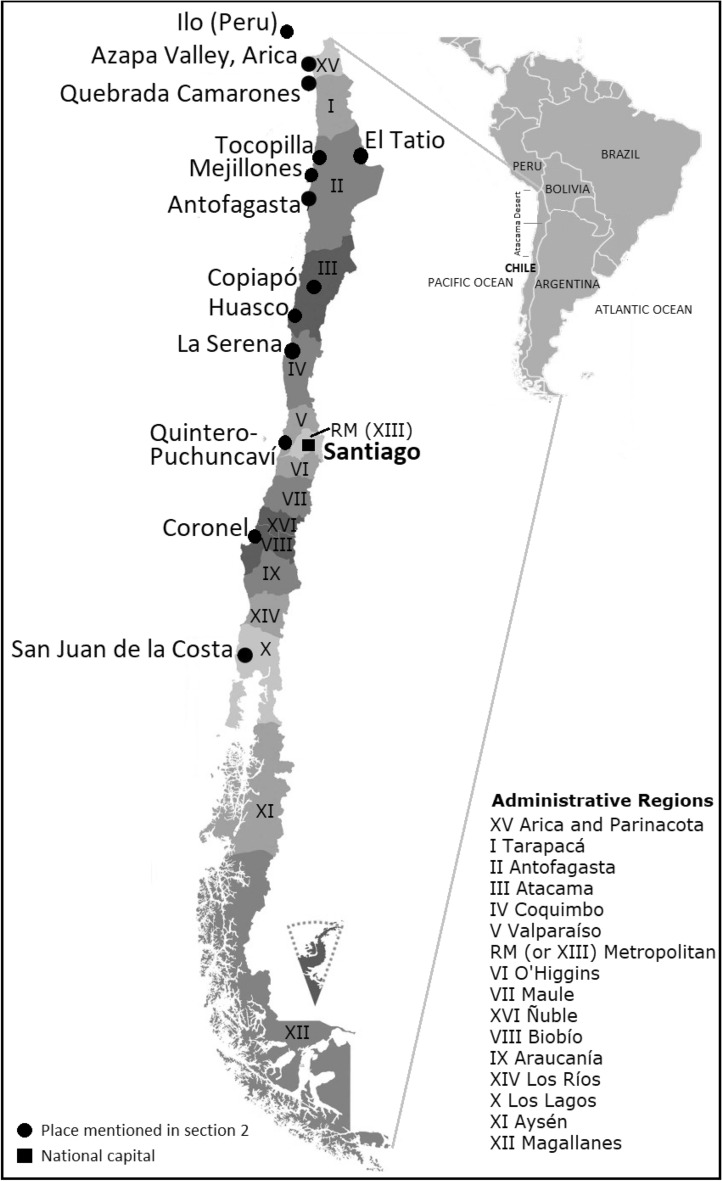
Location of the places (mentioned in the "Arsenic situation in Chile") relevant to As-related studies in Chile (modified from Fig. 2 of Apata et al.^48^, with permission).

Although the first reported case of As-related disease in the Latin American region was from Bell Ville (Argentina), As problem has affected the region since the Precolumbian Era^4^. That is evident from the signs of As ingestion in the mummies of the Chinchorro people living between Ilo in southern Peru and Antofagasta in northern Chile (i.e., 17–22° S, Fig. 2) during 7000–2000 BC^4^. Most of these mummies were discovered in As-bearing mineral-rich zones, where most of the water resources contained high As concentrations (200–5000 μg/L^44–47^). Apata et al.^48^ evaluated whether adverse effects of As on human health, viz. inducing miscarriages, acted as a natural selection pressure that made the Chinchorro population, settled in the Quebrada Camarones (Fig. 2) region of Chile’s Atacama Desert some 7000 years ago, evolve adaptations to it. By observing variations in the gene coding for As methyltransferase (AS3MT) in nearly 150 people from northern (Quebrada Camarones and the Azapa Valley, Arica and Parinacota Region, in the north, Fig. 2) and southern (San Juan de la Costa, Los Lagos Region, Fig. 2) parts of the country, these authors found higher frequencies of the protective variants in people from Quebrada Camarones (68%, Fig. 2), as compared to just 48 and 8% of people in the other two studied areas (Azapa Valley, San Juan de la Costa, Fig. 2). The human body uses the AS3MT enzyme to incorporate As in monomethylarsonic (MMA) acid and dimethylarsinic (DMA) acid^49^. Those who can metabolize As more efficiently convert more of it into the less toxic and more easily expelled DMA^50^. Apata et al.^48^ deciphered a high As metabolization capacity as an adaptive mechanism in these populations to survive in an As-laden environment.

The growing population due to the boom in the mining sector in the mid-twentieth century of the city of Antofagasta (Fig. 2) faced chronic exposure to As between 1958 and 1970, following the inclusion of Holajar (800 µg/L As) and Toconce (1300 µg/L As) rivers as new drinking water sources^51,52^. However, a significant reduction in exposure followed the installation of an As removal plant in 1970, which provided a unique opportunity to study latency effects of exposure to As, carried out by Smith et al.^53^ with mortality data up to 40 years after exposure reduction. These authors concluded that lung, bladder, and kidney cancer mortality due to As exposure have very long latencies, with increased risks manifesting 40 years after exposure reduction; and suggested that As in drinking water may involve one of the most prolonged cancer latencies for a human carcinogen.

Several new scientific studies from As-endemic Antofagasta Region (Fig. 2) in Chile have documented that mortality and morbidity continue to rise even after several decades of efforts to lower the exposure several decades ago^1^. During the last decade, some of the studies highlighted the importance of the timing (age of the person) of As exposure, which can cause severe health issues with significantly higher risk among those exposed to As early in life^54–56^. Another analysis reported a ten-fold increase in the risk of As-associated lung diseases among women exposed to high levels of As in the uterus^57^. Some other studies found that early-life exposure, even at a relatively low dose, increases the risk of As-induced mortality from pulmonary tuberculosis, respiratory problems, and reduced lung function in adults and children^58–61^. Moreover, lifetime and cross-sectional analyses from the Antofagasta Region of Chile (also Mexico) revealed the high risk associated with type 2 diabetes (T2D) hypertension^62–64^. Different studies have described As causing lung, bladder, kidney, and laryngeal cancer, particularly in those patients exposed to As since their birth in the Antofagasta Region^53,57,65–69^. The mortality studies conducted in the same area observed a consistent association with As intake for bladder, laryngeal, and liver cancer among young adults born in Antofagasta, Chile^65–68,70^. Also reported were medical conditions like As methylation, metabolomics, genetic polymorphism, and obesity through drinking water, food chain, and tobacco smoking^64,69^. These findings suggest that interventions targeting early-life As exposure and controlling life lifestyle factors could significantly reduce long-term mortality and morbidity. Khan et al.^1^ have reviewed the effects of early life As exposure on human health, biomarkers, and genetic susceptibility in Latin American countries.

### Sacrifice or saturated zones

Smith et al.^65^ found marked increased mortality from many cancers and other diseases between 1950 and 2000 in the Antofagasta Region. Later, Smith et al.^53^ presented Poisson regression rate ratios (RRs) for lung, bladder, and kidney cancer and acute myocardial infarction mortality for the same region in comparison with the rest of Chile from 2001 to 2010. They found that lung and bladder mortality were greatly elevated (RR = 3.38, with 95% confidence interval [CI] 3.19–3.58, P < 0.001 for lung cancer in men; RR = 2.41, with 95% CI 2.20–2.64, P < 0.001 for lung cancer in women; RR = 4.79, 95% CI 4.20–5.46, P < 0.001 for bladder cancer in men; RR = 6.43, with 95% CI 5.49–7.54, P < 0.001 for bladder cancer in women), together with kidney cancer mortality (RR = 1.75, with 95% CI 1.49–2.05, P < 0.001 for men; RR = 2.09, 95% CI 1.69–2.57, P < 0.001 for women). They further compared RRs from the Antofagasta Region with the Valparaiso Region (Fig. 1) for all the years from 1950 to 2010, considering the Valparaiso Region unexposed to As in their study. However, on the contrary, this region is one of the most contaminated ones, with Quintero-Puchuncaví (Fig. 2) being one of the most polluted zones in the country known for a long time^71^. It is one of the so-called “sacrifice zones” in the country. Terram^72^ prepared a series of fact sheets describing the so-called "Sacrificial Zones" in Chile. These zones are environmentally devastated national territories due to industrial development, such as the communes of Tocopilla and Mejillones (Region of Antofagasta, Fig. 2), Huasco (Region of Atacama, Fig. 2), Quintero-Puchuncaví (Region of Valparaíso, Fig. 2) and Coronel (Region of Biobío, Fig. 1). Lerner^73^ used the term “sacrifice zones” for the areas with thousands of residents exposed to disproportionately elevated levels of hazardous chemicals. It is derived from the Orwellian term "National Sacrifice Zones", coined in the erstwhile Soviet Union to describe populated areas irrevocably polluted by nuclear fallout, because of mining and processing of uranium into nuclear weapons, during the Cold War with the United States^73^. However, socio-ecological movements in Chile use this term to characterize geographical areas where industrial waste disposal is so much that the entire area and its population are considered sacrificed on the altar of economic growth. In this context, the opening of the Ventanas Industrial Complex (VIC^74–77^) at the Quintero-Puchuncaví Bay was justified, based on the appeals to patriotic feelings, urging the people to accept "some sacrifices" for the country's progress. On July 17th, 1957, the editorial of the Chilean Newspaper El Mercurio de Valparaíso wrote, "People must approach this problem with patriotism, and they must accept some sacrifices; otherwise, establishing a smelting plant would not be possible in any place in the country. The industrialized nations have accepted these sacrifices. It is the price of progress. Rain is indispensable for agriculture, but when it rains, someone must get wet" (translated from Spanish). The official recognition of this contaminated zone is in terms of the declaration of VIC as a "saturated zone" through a decree^78^. In his annual message of 21 May of 2012, erstwhile Chilean President Sebastián Piñera referred to this situation by saying that "… the environmental and health tragedies of Ventanas, Huasco, Coronel and Mussels speak better than a thousand words. Chile does not deserve this"^79^. In a significant move, Chile's state-owned and the world's largest copper producer Codelco (Spanish acronym for the National Copper Corporation of Chile, previous owner ENAMI: National Mining Company; Fig. 2) closed its VIC metal smelter at Quintero, located some 108 km (67 miles) northwest of the capital, Santiago. The decision to shut down was taken in October 2022 after authorities declared an environmental emergency due to pollution that left dozens suffering from symptoms of sulfur dioxide emission poisoning^80^.

It is not clear on what basis Smith et al.^53^ considered the Valparaiso Region free of exposure despite the contamination problem in the region being well-recognized for a long time^71^. Near VIC, which has at least 12 high-impact industries operating in an area of 8.5 km^2^, including oil refineries, chemical processing units, thermoelectric plants, and a smelting plant^81^, a soil mapping survey found the concentrations of As over 40 mg/kg, with a maximum of 824 mg/kg close to this complex, and median concentration of 13.4 mg/kg^82^. The background As value determined around VIC with median absolute deviation method (31.6 mg/kg^82^); is much higher than reported median As concentrations in urban soils of Arica (median value 17.4 mg/kg^83^, Fig. 2) or Copiapó (28.5–36.8^84,85^, Fig. 2), in recognizably more contaminated northern Chile.

Rueda-Holgado et al.^86^ compared elemental deposition of toxic elements near VIC with reported values in other areas of the world. They found concentration at La Greda elementary school higher than the Aliaga industrial region in Turkey^87^. Reported atmospheric deposition values for As, Copper (Cu), Zinc (Zn), Antimony (Sb), and Iron (Fe) at La Greda were higher than two large urban areas, namely, Venice in Italy^88^ and Belgrade in Serbia^89^.

Although VIC is recognized as a "sacrifice zone"^90–95^ by environmental activists, researchers and the general public or "saturated zone"^78^ by the government, there are potential naturally contaminated zones in the country, which are saturated but unrecognized—considering the extent of metallogenic belts across the country and the presence of associated mining and mineral processing industries that accelerates and augments natural As-release and dissemination into human environments.

## Principal geogenic As sources

### Arc magmatism and related geothermal activities

Arc magmatism and associated geothermal activity contribute to As contamination of the aquifers through in situ leaching^96,97^. Moreover, high rates of erosion due to rapid uplift of the arc zones and subsequent transport of the eroded sediments to the downstream basins lead to the presence of As-bearing minerals in arc derived sediments in the aquifer matrix^96,97^. The reported As concentrations in various lithologies worldwide suggest a close association with magmatic arcs at convergent continental margins (e.g., the Andes) and collision belts (e.g., the Himalayas) that inevitably contain arc-derived components^96–105^. Accordingly, high As content in arc magmas originates from one or more of the following: (a) the subducting oceanic crust with its sediment cover; (b) the mantle component of the subducting slab; (c) the mantle wedge component overlying the slab; (d) the overlying continental crust through which the arc magma ascends to the surface^106–108^.

Arc volcanism in the Andes leads to the emission of As-laden volcanic ash up to 10 mg/kg As^109^ that can be dispersed in the air to eventually settle down or carried in suspension or solution to the downstream basins. Arsenic enriched rhyolitic glass in volcanic ash undergoes hydrolytic dissolution and produce an influx of major (e.g., Na^+^, K^+^, Si^4+^, and HCO_3_^−^) and minor (e.g., oxyanions of As, V, and Mo) solutes to groundwater under conducive climatic conditions^109^.

Most of the high-enthalpy Latin American geothermal reservoirs are found at various depths in active volcanic systems between southern Chile and northern Mexico and within the Trans-Mexican volcanic belt. However, there exist geothermal systems not associated with volcanism. For example, Alam et al.^110,111^ classified geothermal systems along the Liquiñe Ofqui Fault Zone (LOFZ) in south-central Chile into two domains, the first one associated with volcanism (e.g., Palguín, Coñaripe) and the other one with a deep circulation of meteoric water (e.g., Liquiñe, Chihuío).

#### Volcanism

Of the four Andean volcanic zones^112^, three belongs to the Chilean Andes, namely, the Central (CVZ), Southern (SVZ), and Austral (AVZ) Volcanic Zones, separated by the Pampean Flat Slab (PFS; 27°–33° S) segment and Patagonian Volcanic Gap (PVG, 46°–49° S), respectively. The absence of volcanism and geothermal activities in PFS is a consequence of the low subduction angle that prevents the formation of an asthenospheric wedge from which magma could be generated in the Earth's mantle^112^. On the other hand, several workers^112–114^ attributed the absence of volcanism in the PVG to a slab window, as without slab, there is no arc magmatism.

The most recent list of Chilean volcanoes ranking, based on the specific associated risk, by SERNAGEOMIN (Chile’s National Geology and Mining Service^115^) includes 92 active volcanoes, with the most highly ranked ones belonging to SVZ. According to this ranking, of the 14 volcanoes with the highest associated risk and recent volcanic activities above volcanic explosive index (VEI^116^) 4, all but Láscar (Antofagasta Region) are in southern Chile. Because of the highly explosive nature (mostly Plinian^115^) of these volcanoes, pyroclastics emission is vast. Among the ejected tephra, volcanic ash travels intercontinental distances. For example, the eruption of Puyehue-Cordón Caulle not only disrupted the air traffic in Argentina but as far away as Australia and New Zealand^117^.

Unlike northern Chile's predominantly mining-based economy and desert climate, southern Chile is characterized by agriculture and agroindustry due to its Mediterranean and temperate climate. Although volcanic ash has been considered suitable for the soil in vineyards and other fruit production because of the nutrient elements (e.g., Chile’s Maule and Colchagua Valleys, USA’s Napa and Willamette valleys, Sonoma, and Lake counties)^118^, there has been no attention towards toxic elements, viz. As, contributed by volcanic ash to the soil. The volcanic ash's vitric content accumulates elevated As concentration, which gets easily dissolved due to its amorphous nature during transport in the water or leached from aquifer matrix in the plains. This phenomenon is well-recognized in the Chaco-Pampean Plain in Argentina. Bundschuh et al.^119^ reported volcanic ash layers with 90% of rhyolitic glass and volcanic glass dispersed in the sediments, along with the clastic sediments of metamorphic and igneous origin, as potential groundwater As sources in a case study from Robles county, Santiago del Estero Province (Argentina) in this plain. Nicolli et al.^120^ considered volcanic glass dissolution and/or hydrolysis and leaching of silicates minerals hosted in loess one of the three key processes rendering high As concentrations in shallow aquifers. On the other hand, Botto et al. showed adsorptive capability of soil by iron activation^121,122^ using two representative samples (average composition and Fe-rich materials) of the volcanic ash from Puyehue Cordon Caulle Volcanic Complex (Chile), emitted on June 4, 2011, and deposited in Villa La Angostura ∼ 40 km from the source^122^.

#### Geothermal activities

Geothermal systems associated with these volcanic centers potentially contaminate the groundwater, where the ascending As-enriched geothermal fluids meet the aquifers. Geothermal activity is controlled mainly by volcanism and regional geologic structures, viz. Toculla-Puchuldiza Fault in northern Chile^123^, Infiernillo and San Ramón-Pocuro fault systems in central Chile^124^, Liquiñe-Ofqui fault system in southern Chile^110,111,125–130^. The CVZ of Chile lies between latitude 14° S and 27° S^112^. The geothermal fields in CVZ (between 14° and 27° S^112^) at high elevations (> 3000 m above mean sea level: amsl) are structurally controlled by N–S and N–W-trending grabens^131^, besides volcanic activity. In these geothermal fields, Lower Miocene–Pleistocene ignimbrite deposits and andesitic–rhyolitic volcanics overly Middle Cretaceous-Upper Miocene volcano-sedimentary sequences^123,131^. All these lithologies are important As sources with their elevated concentration in volcanic rocks range between 1.0 and 3105 mg/kg (median 7 mg/kg^10,11,13,14^).

An inventory of over 150 Chilean hot springs across the country, prepared by Hauser^132^, was updated by Risacher and Hauser^24^ and used by Risacher et al.^25^ to evaluate the origin of the thermal waters. Pérez^133^ described most of the southern hot springs between 39 and 42° S in south-central Chile. While Tassi et al.^123,134–136^ and Aguilera et al.^137,138^ carried out gas geochemistry of volcanic and hydrothermal fluids in northern Chile, Ray et al.^139^ studied gas and isotope geochemistry of hydrothermal fluids in southern Chile. Some of the most extensively studied geothermal areas include El Tatio^134,140–143^. Specific studies in other areas are scarce. Mahon and Cusicanqui^144^ described Puja geothermal field, apart from Puchuldiza, in northern Chile. Sepúlveda et al.^145,146^ examined one of the largest active geothermal systems of southern Chile, Puyehue-Cordón Caulle.

**Figure 3:**
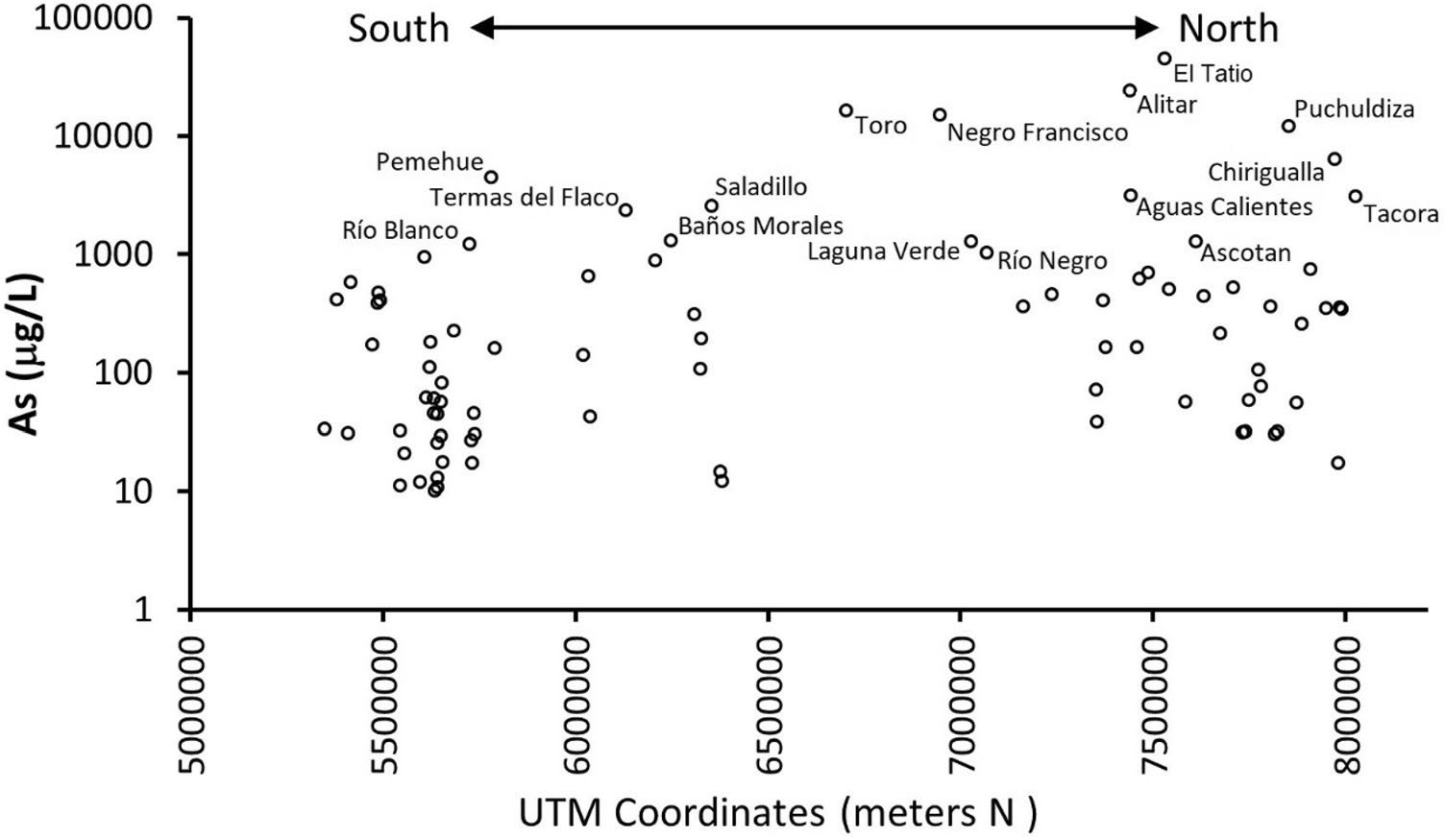
Variation of Arsenic (As) concentration in thermal waters of Chile (UTM Coordinates WGS 84/zones 18, 19S) with the locations where As concentration is above 1000 µg/L.

While the highest reported As concentrations (> 10,000 μg/L) are from five geothermal fields, namely El Tatio, Alitar, Toro, Negro Francisco, and Puchuldiza, in northern Chile, reported concentrations between 10 and 999 μg/L are for the hot springs evenly distributed in northern and southern Chile (Table 1, Fig. 3).

**Table 1:** Arsenic (As) concentration in thermal waters of Chile^24^ (UTM Coordinates WGS 84 / zones 18, 19S).

As range (μg/L)	Hot spring	Administrative region	UTM			Altitude (m)	Temp (°C)	pH	TDS (mg/L)	As (μg/L)	Geographic region
			zone	E	N						
> 10,000	Puchuldiza	I	19 K	504,281	7,853,446	4209	87	7.38	4526	11,950	Northern Chile
	El Tatio	II	19 K	602,331	7,530,506	4268	82	6.73	14,364	45,300	Northern Chile
	Alitar	II	19 K	637,318	7,439,332	4708	63.2	5.65	1820	23,900	Northern Chile
	Negro Francisco	III	19 J	491,796	6,946,892	4500	17.7	6.65	9719	15,000	Northern Chile
	Toro	IV	19 J	402,775	6,702,743	3484	54.4	6.73	5381	16,400	Northern Chile
1000–9999	Tacora	I	19 K	412,889	8,040,771	4501	40.6	2.05	4247	3580	Northern Chile
	Chirigualla	I	19 K	481,487	7,971,793	4461	46.6	6.83	3809	6330	Northern Chile
	Ascotan	II	19 K	577,586	7,610,918	3728	21	7.15	3768	1270	Northern Chile
	Aguas Calientes	II	19 K	661,102	7,441,754	4226	49.8	6.61	21,753	3150	Northern Chile
	Rio Negro	III	19 J	518,123	7,067,954	4100	40.2	7.66	2320	1020	Northern Chile
	Laguna Verde	III	19 J	551,178	7,028,028	4355	40.5	7.52	3884	1270	Northern Chile
	Saladillo	V	19H	381,295	6,353,630	1765	24.7	6.57	3628	2570	Central Chile
	Baños Morales	RM	19H	407,534	6,247,777	2374	23	6.31	27,231	1310	Central Chile
	Termas del Flaco	VI	19H	368,693	6,130,552	1716	55.1	6.83	2153	2330	Central Chile
	Pemehue	IX	19H	262,959	5,782,067	768	38	7.05	2933	4460	Southern Chile
	Río Blanco	IX	19H	271,781	5,727,019	1288	81.3	4.64	198	1220	Southern Chile
100–999	Las Cuevas	I	19 K	454,421	7,990,956	4487	32.2	6.99	1460	345	Northern Chile
	Jurase	I	19 K	446,034	7,986,486	4053	64.9	7.04	2147	357	Northern Chile
	Chitune	I	19 K	449,923	7,948,523	3555	33.9	9.04	830	350	Northern Chile
	Surire	I	19 K	500,105	7,909,346	4262	49	6.09	4826	749	Northern Chile
	Parajalla	I	19 K	509,466	7,885,769	4269	29.4	6.81	1271	256	Northern Chile
	Pampa Lirima	I	19 K	509,736	7,804,814	3994	66	6.19	1284	360	Northern Chile
	Macaya	I	19 K	481,849	7,774,010	2750	31	8.38	417	106	Northern Chile
	Uruputuncu	I	19 K	542,873	7,708,130	4021	36	2.43	7433	521	Northern Chile
	Alconcha	II	19 K	553,482	7,673,910	4185	43.4	6.46	893	213	Northern Chile
	Carcote	II	19 K	560,125	7,631,200	3691	24.2	7.08	7891	442	Northern Chile
	Turi	II	19 K	574,066	7,541,583	3088	22.1	5.83	2016	506	Northern Chile
	Puritama	II	19 K	598,217	7,487,190	3535	33.3	7.09	1857	700	Northern Chile
	San Pedro Pozo 3	II	19 K	585,700	7,464,575	2430	25	7.39	2422	622	Northern Chile
	Tara	II	19 K	673,985	7,457,325	4320	19.5	6.94	745	164	Northern Chile
	Miscanti	II	19 K	627,574	7,377,232	4147	35.8	8.03	675	163	Northern Chile
	Tilopozo	II	19 K	577,705	7,369,627	2309	26.3	6.93	2757	408	Northern Chile
	Aguas Calientes	II	19 K	537,930	7,237,990	3668	24.3	6.83	1685	459	Northern Chile
	Salar de la Isla	III	19 J	536,695	7,163,035	3952	26.4	7.79	6263	360	Northern Chile
	Colina Pedehue	RM	19H	351,014	6,327,245	948	29	7.82	443	193	Central Chile
	Carvajalino	RM	19H	349,984	6,326,017	813	22.9	7.84	371	107	Central Chile
	Tupungato	RM	19H	415,984	6,308,513	2983	28.3	6.13	3765	311	Central Chile
	Cauquenes	VI	19H	356,709	6,209,255	759	48.9	8.5	3407	883	Central Chile
	Medano	VII	19H	340,983	6,034,212	984	32.5	7.18	1061	653	Southern Chile
	Campanario	VII	19H	356,931	6,022,173	1596	51.2	5.83	18,554	141	Southern Chile
	Avellano	VIII	19H	277,709	5,792,134	620	62.3	8.53	839	161	Southern Chile
	Balboa	IX	19H	265,222	5,684,573	850	66.5	6.79	871	225	Southern Chile
	Geométricas	X	19H	252,841	5,623,697	905	64.7	7.84	744	179	Southern Chile
	Vergara	X	19H	251,383	5,622,753	772	45.2	8.36	478	111	Southern Chile
	Coñaripe	X	19H	249,121	5,608,668	253	62	7.66	437	942	Southern Chile
	Pangal	X	19H	729,046	5,493,850	228	35.2	7.56	937	404	Southern Chile
	Puyehue	X	19H	725,795	5,489,751	347	48	7.85	440	469	Southern Chile
	Aguas Calientes	X	19H	727,359	5,487,111	471	62	6.92	636	385	Southern Chile
	Rupanco	X	19H	734,079	5,473,194	215	63	7.4	644	171	Southern Chile
	Ralun	X	19H	723,824	5,417,854	110	43	7.02	2940	577	Southern Chile
	Puelo	X	19H	722,951	5,382,193	32	35.2	6.44	4472	415	Southern Chile
10–99	Pozo Rio Lauca	I	19H	464,168	7,980,123	4380	21.5	8.26	150	17.4	Northern Chile
	Enquelga	I	19H	521,865	7,873,169	3869	30.4	6.14	1821	55.7	Northern Chile
	Chuzmiza	I	19H	480,804	7,823,444	3377	42.5	8.16	624	32	Northern Chile
	Guasquina	I	19H	458,465	7,816,762	2000	27.5	8.48	425	30.2	Northern Chile
	Mamina	I	19H	477,924	7,780,502	2802	51.6	8.61	480	76.9	Northern Chile
	Puquio La Calera	I	19H	459,221	7,748,539	1390	31.8	7.78	2092	58	Northern Chile
	Pozo Essat	I	19H	446,143	7,739,834	990	27.3	7.65	1281	32	Northern Chile
	Fuente Santa Rosa	I	19H	462,980	7,733,912	1250	28.8	8.05	787	31	Northern Chile
	Taira	II	19H	542,004	7,584,400	3188	28.2	5.99	2241	56.1	Northern Chile
	Capur	II	19H	624,985	7,353,987	3955	22.8	7.57	6675	38	Northern Chile
	Tuyajto	II	19H	644,410	7,353,468	4039	32.6	6.45	2252	71.5	Northern Chile
	Jahuel	V	19H	350,574	6,382,665	1172	21.2	7.14	548	12.1	Central Chile
	El Barro	V	19H	355,068	6,376,511	1086	18.8	7.58	497	14.5	Central Chile
	Paninavida	VII	19H	281,575	6,039,788	204	30.7	9.91	382	42.2	Southern Chile
	Manzanar	IX	19H	263,912	5,739,326	755	52	9.78	261	30.3	Southern Chile
	Malalcahuello	IX	19H	274,737	5,736,701	962	41.5	9.24	199	45.3	Southern Chile
	Alaska	IX	19H	289,379	5,733,249	925	30.4	8.54	478	17.4	Southern Chile
	Celis	IX	19H	258,103	5,730,171	691	24.2	8.88	125	26.8	Southern Chile
	Quimey-Co	IX	19H	267,264	5,655,408	491	42.4	7.87	240	17.5	Southern Chile
	Los Pozones	IX	19H	271,455	5,654,872	588	53.2	7.91	301	81.1	Southern Chile
	Panqui	IX	19H	281,669	5,652,080	961	49.7	7.57	369	55.9	Southern Chile
	Liucura	IX	19H	259,081	5,650,777	328	28.8	7.97	257	29.3	Southern Chile
	Menetue	IX	19H	265,473	5,643,208	386	43.9	8.23	291	25.5	Southern Chile
	Ancamil	IX	19H	277,367	5,642,316	395	32.7	7.38	267	44.4	Southern Chile
	San Luis	IX	19H	268,077	5,642,034	407	35.8	8.27	239	13	Southern Chile
	Trancura	IX	19H	267,730	5,642,020	430	34.2	7.73	239	10.7	Southern Chile
	Lahuenco	IX	19H	280,952	5,635,954	398	27.4	8.24	257	10.1	Southern Chile
	Rinconada	IX	19H	270,078	5,633,141	661	29.1	6.84	787	45.1	Southern Chile
	Palguin	IX	19H	260,157	5,632,793	783	43.7	8.52	271	60.7	Southern Chile
	Culan	X	19H	248,731	5,614,027	357	33	8.36	268	61.2	Southern Chile
	Quintoman	X	19H	256,267	5,597,069	268	62.7	8.92	295	11.9	Southern Chile
	Cerrillo	X	19G	743,072	5,557,033	361	40.3	9.05	245	20.9	Southern Chile
	Chihuió	X	19G	250,204	5,546,584	336	81.5	8.72	431	11.1	Southern Chile
	Llifen	X	19G	733,359	5,545,890	187	18	8.32	144	32.5	Southern Chile
	Rollizos	X	19G	723,836	5,410,466	14	28.3	7.65	1291	30.8	Southern Chile
	Pichicolo	X	19G	703,000	5,350,466	96	37	7.66	281	33.5	Southern Chile

##### El Tatio geothermal field

In the Antofagasta Region, El Tatio geothermal field (ETGF), known for its geysers located at an elevation of 4300 m amsl, has reported As concentration up to 50 mg/L in the surface discharges^24,27,132,147^. The isotopic data on the geothermal discharges of ETGF indicate an essential role of water–rock interactions^141^ and mixing between meteoric water, carbon dioxide (CO_2_) hydrogen sulfide (H_2_S), and/or hydrocarbon-rich magmatic fluids^134^ in establishing geochemistry of the hydrothermal fluids. Studies of solid-state partitioning in this geothermal area's sediments have shown that As is mainly associated with carbonates^148^.

Several authors have identified ETGF as the principal natural As source for the Loa River, the longest (440 km) river of Chile, mainly contributed through its tributary Salado, fed by ETGF waters^30^. Close to ETGF, As content in Salado River sediments is ~ 11,000 mg/kg and ~ 700 mg/kg at the Loa river's mouth, where it discharges into the Pacific Ocean (Romero et al.^30^). Due to dilution and adsorption of As onto solid materials, the concentration of As in Salado River water decreases downstream to 1.2 mg/L at its confluence with Loa River, and then to ~ 0.1 mg/L at latter's mouth at the Pacific Ocean^30,149^. Romero et al.^30^ used sequential extraction analyses to show that As is mainly associated with Iron and Manganese (Fe–Mn) oxy-hydroxides and residual phases; however, part of the As (about 20%) is readily available, extracted from the exchangeable and carbonate phases. This result is in agreement with the correlation observed between As content in sediments and As concentration in waters in the area^148^. The extreme arid conditions, high evaporation, and the lack of low-As tributaries that could have diluted the significant As input by Salado tributary contribute to maintaining high As concentration in the Rio Loa water to the mouth. Due to the oxidizing conditions, neutral to alkaline pH, high salinity, and high As concentrations, adsorption of As-species is not favored^30^. Although the main As source in the Loa River basin is geogenic, smelter emissions and mining wastes and the As-rich effluents from the water treatment plants also contribute to As contamination^30^.

To address the problem of determining the relative contribution of contaminants to the Loa River from ETGF and the mines using spatial patterns in trace metal/metalloid concentrations, Wilson^148^ used selected isotopic tracers, including ^121^Sb and ^123^Sb to assess contaminant provenance. Thus, ETGF is the source of As and other toxic elements for downstream users across the Antofagasta Region^148,150,151^. Landrum et al.^151^ also reported that As (∼ 0.45 mmol/L) and Sb (0.021 mmol/L) concentrations at ETGF are the highest reported for natural surface water. The ETGF waters are near neutral Sodium-Chloride (Na-Cl) type with As and Sb primarily in the reduced (III) redox state at the discharge with progressive oxidation downstream. Landrum et al.^151^ also found that the Fe(III) oxide-hydroxide or ferric (Fe^3+^) oxyhydroxide associated with the microbial mats and some mineral precipitates accumulate substantial As, identified as arsenate by X-ray absorption spectroscopy analysis (> 10 wt% in the mats). This As is easily mobilized by anion exchange or mild dissolution of hydrous Fe (ferric) oxide The ubiquitous microbial mats represent a significant reservoir of As in this system. Landrum et al.^151^ reported As concentration in silica sinters up to 274 mmol/kg.

Alsina et al.^26,152^ further identified hydrous ferric oxides as the main As-bearing phase in sinter from this geothermal field, suggesting sorption as the primary mechanism for As scavenging by the solid phases (sinters). Besides, they identified nodular arsenide micro-mineralizations, like Loellingite (FeAs_2_), during the bulk-scale analysis of the sinter material. Based on the presence of arsenide mineralizations, these authors indicated the development of anoxic environments on the surface of the siliceous sinter, thus suggesting more complex biogeochemistry for As than usually observed for circum-neutral pH brine geothermal environments.

Nicolau et al.^15^ reported a rare As borate Cahnite (Ca_4_B_2_As_2_O_12_∙4H_2_O) and other unidentified Calcium (Ca)–As–Fe-rich minerals with needle-like and flower-like crystals, detected by both XRD and SEM in the colored sinter samples the sinters from ETGF. Besides, these authors also found other As minerals, viz. Teruggite (Ca_4_MgAs_2_B_12_O_28_⋅20H_2_O) and Nobleite (CaB_6_O_10_⋅4H_2_O), documented previously, together with other mineral phases, viz. Halite, Gypsum^153,154^. The very soluble As and Boron (B) minerals precipitate by complete evaporation of B and As-enriched waters^15^. Moreover, these authors highlighted the particularity of the thermal waters and precipitates (sinters) of ETGF, e.g., As and B contents higher than that of Cistern Spring, Champagne Pool, and Reykjanes, and the presence of As and B minerals: Cahnite, Sassolite, Nobleite, and Teruggite.

Nicolau et al.^15^ further observed that the sinter deposits around the group of less diluted thermal springs do not show higher content of accessory minerals (e.g., Realgar, Orpiment), indicating no direct relation between the concentration of these elements in the currently discharging thermal waters and the abundance of Halite, Gypsum, and Cahnite in the sinter deposit. According to these authors, this difference between the water and sinter chemistry could be because some sites do not reach full evaporation states, necessary for this highly soluble minerals’ precipitation. Thus, Sodium (Na), Ca, B, and As largely remain in the fluid phase. They observed reddish coloration of the analyzed sinter samples and attributed it to the occurrence of Ca–As–Fe-rich flower-like crystals between the Silica layers, as they constitute the red porous friable laminations in some sinter samples.

##### Puchuldiza geothermal field

The Puchuldiza geothermal field (PGF), located in the Tarapacá Region of northern Chile at an elevation of 4200 m amsl, has surface manifestations over an area of ~ 1 km^2^^16^. The geothermal activity at PGF is controlled by NW–SE reverse fault and NNE–SSW normal strike-slip fault systems comprising of the Churicollo, Puchuldiza, and Tuja faults^123^. Sanchez-Yanez et al.^16^ further provided in situ trace element data, including As (up to 60,189.18 mg/kg), in metal-rich silica sinter samples from the Puchuldiza geothermal field in the Chilean Altiplano. This silica sinter has complete diagenetic sequence, from non-crystalline Opal A to microcrystalline Quartz, wherein As is predominantly enriched in the more amorphous silica phases (Opal-A/Opal-CT). Moreover, it contains As-bearing accessory minerals (e.g., Realgar, Orpiment) and Fe-oxyhydroxides that efficiently adsorb As control its incorporation. These authors further reported that no B-bearing minerals were found at Puchuldiza, unlike El Tatio, where Nicolau et al.^15^ reported As borate Cahnite (Ca_4_B_2_As_2_O_12_·4H_2_O) in sinter samples.

##### Other geothermal areas

The central portion of the Pampean Flat Slab (PFS, between 27° and 33° 30′ S^112^) is the Andes' most elevated segment^155^. Although there is an absence of volcanism in PFS, there exist geothermal systems with surface expressions in the form of 17 thermal springs documented by Hauser^132^ and Risacher and Hauser^24^. Arsenic concentration in Toro hot spring, located at the northern limit of the PFS at an elevation of approximately 3500 m amsl in Coquimbo Region, is 16.2 mg/L^24,132^.

Volcanism and geothermal activities in SVZ, extending the subducting Juan Fernández Ridge to the Chile Rise^112^, are controlled by regional fault systems, namely the NNE Liquiñe Ofqui Fault System (LOFZ^123,154–157^ and the WNW Andean Transverse Faults (ATF^123,158^). Thermal waters from 120 hot springs documented by Hauser^132^, Pérez^133^, and Risacher and Hauser^24^ in SVZ geothermal fields, in general, have relatively lower As concentration than CVZ, ranging from 0.003 to 6.127 mg/L with an average of 0.331 mg/L^22^. These authors associated the lower values of As in the SVZ to the lower crust thickness (30–40 km) and higher precipitation rates, which could dilute As concentrations at the surface.

### Metallogeny and metallurgical processes

**Figure 4:**
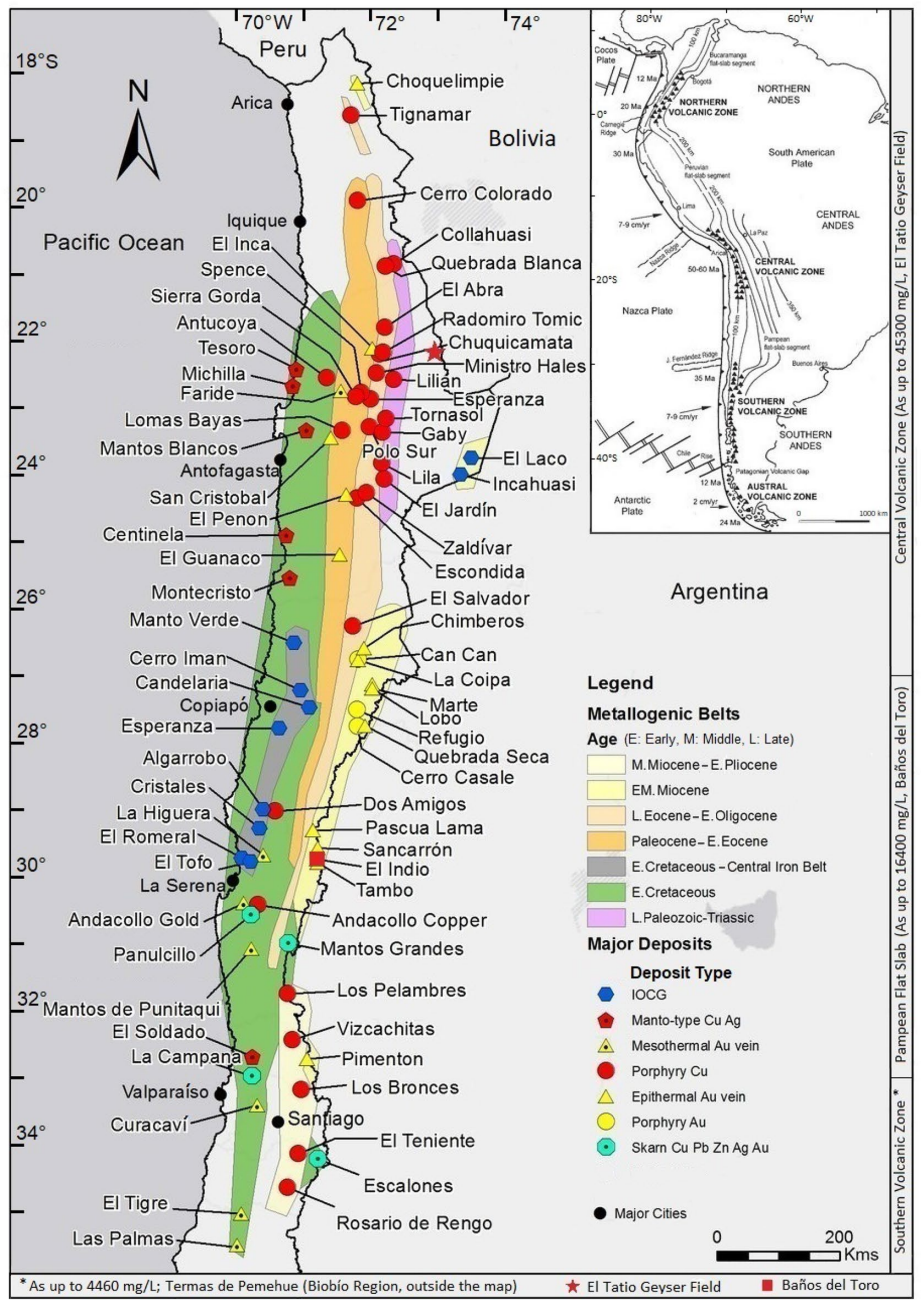
Location of Chilean metallogenic belts with the location of the major deposits and principal cities (source: Kura Mineral Resources website, kuraminerals.com/chile-mining, used here with permission) and the highest As concentrations^24^ in different segments of the Andes, proposed by Stern et al.^112^. See the inset map^112^ (reproduced with permission) for the details of the tectonic elements and varying depth in kilometers to the Benioff zone, shown by the contours.

Major metallogenic belts are confined to northern and central Chile until ~ 35° S (Fig. 4), which marks a primary difference between the southern and northern-central regions. The Lower Eocene–Early Oligocene porphyry copper belt is having elevated As concentrations in the copper concentrates (e.g., 0.79% As at Chuquicamata^159^). Also, epithermal deposits along the eastern border of northern Chile are known for the presence of As-rich minerals, viz. Enargite (Cu_3_AsS_4_) and Tennantite (Cu_12_As_4_S_13_), in copper concentrates (e.g., 8% As at El Indio^18,19^). In southern Chile, there are only a few polymetallic deposits exploited in the recent past, e.g., El Toqui mine in the Aysén Region. Other metal deposit types present in this part of the country have not been exploited or studied in detail, viz. Arsenopyrite bearing placer gold deposits in Tierra del Fuego area of the Magallanes Region. In the Coastal Range of south-central Chile, between 38 and 42° S latitudes, there are ore occurrences related with a structurally dismembered Paleozoic ophiolitic complex^160^. Within this ophiolite suite, there are massive polymetallic sulfides interbedded with metabasites ("greenschists"^161^), which have been interpreted as volcanic-exhalative depositions formed on the oceanic bottom during the Paleozoic. These ore occurrences are genetically like Japan's Besshi-type deposits, characterized by their spatial relationship with tholeiitic volcanic and volcaniclastic rocks. Moreover, reported placer gold deposits along the beaches in the southernmost Aysén and Magallanes regions^162^ indicate the gold mineralized zones down South.

Although the distribution of geogenic As contamination around localized ore bodies is generally limited and defined largely by geotectonic setting prevalent at the time of their formation, anthropogenic materials associated with ore mining and processing, viz. tailings and sludge, expand the area of contamination into the hydrosphere, thus affecting surface and groundwater, and pedosphere, thus affecting soil horizon and near-surface ecosystems^163^. Moreover, chemical weathering of the excavated ore bodies, exposed to exogenic forces of weathering and erosion, produce As-contaminated sediments, which are the potential sources of As contamination of the environment and consequent human As exposure^163^. Mining and metallurgical activities cause proliferation of As contamination to air, sediments, and streams, which might remain contaminated long after the cessation of these activities.

Concentrates generated from porphyry copper deposits in Chile present elevated concentrations of As, e.g., Chuquicamata (7900 mg/kg or 0.79% As^159^), El Indio (80,000 mg/kg or 8% As^19^). Arsenic is mainly related to Tennantite (Cu_12_As_4_S_13_) and Enargite (Cu_3_AsS_4_), which diminish the economic value of the concentrate^164^. Tailing impoundments sampled by SERNAGEOMIN^165^ show that the highest values of As within solid materials reach up to 60%. In the following paragraphs, we present some specific cases of As contamination associated with smelting plants.

Case 1: Pyrometallurgical processes at the Paipote National Foundry of the National Mining Company (ENAMI), operating since 1951, produce toxic emissions—gases, volatile elements, and microparticles—expelled directly into the atmosphere in the Paipote sector of the Copiapó River Valley^166^. The toxic microparticles are transported by winds and subsequently deposit in the soils of the surrounding area. The heavy metals identified in these soils through the spectral diagrams (qualitative SEM analysis) are As, Lead (Pb), Chromium (Cr), Fe, Cadmium (Cd), Cu, Molybdenum (Mo), and Zinc (Zn).

Case 2: At VIC (see "Sacrifice or saturated zones"), students at La Greda School, located 500 m to the east from the smelting plant currently belonging to Codelco (previous owner ENAMI) were reported to have a considerable amount of As and Pb in blood^167^, although absolute values were not reported.

Case 3: The reported average As concentration in the proximity of Potrerillos smelter in the northern Atacama Region is 730 µg/L and 463 mg/kg in water and sediment, respectively. Mine wastes produced by Potrerillos and El Salvador were discharged directly into the El Salado Rive without any treatment until March 1990, following a court ruling related to this contamination^168^. However, by the time this practice was discontinued, up to 9 m thick tailing material deposited along the Chañaral coast for 5 km (N–S) with lateral spread (E–W) varying from 0.4 to 0.88 km by 5 km^169^. Of 120 grid (250 m × 250 m) sediment samples over 4 km × 3 km grid in Chañaral, Fernández and Tapia^170^ found As and Cu content exceeding the Canadian standard in 90%, and US and Brazilian standard in 60% of the samples.

### Soil

Based on the analysis of 26 soil samples from three different Chilean administrative regions, De Gregori et al.^171^ reported high As and Sb concentrations in all soils from northern Chile, especially at Calama and Quillagua, in the Loa River Valley. They attributed it to the Salado River's contaminated irrigation waters, originating in the geothermal zone El Tatio (see "El Tatio geothermal field"). Another study in the same basin by Bugueño et al.^172^ reported differential As binding in the sediments at two sites. Oyarzún et al.^19^ reported high As concentrations (747 ± 544 mg/kg, n = 14) in Holocene (BP 9640 ± 40) lacustrine sediments near the El Indio gold deposit, in which As is mainly related to iron oxides and oxyhydroxides. The available data on As shows its natural presence in rocks (1.0 and 3105 mg/kg, median 7 mg/kg), soils and sediments (1.6 to 2886 mg/kg, median 18 mg/kg) in Arica and Parinacota, Tarapacá, Atacama, and Coquimbo administrative regions^10,11^, as well as in saline precipitates^13,14^ and marine sediments^173^. Arsenic content in the samples of the mud spread throughout the city of Copiapó following the flooding of the Copiapó River in April 2017, and airborne dust of the dried mud was about 5 mg/kg^174^. In the following paragraphs, we present some studies of As contamination of soil in Puchuncaví and Catemu valleys associated with VIC in Quintero- Puchuncaví area of Valparaíso Region.

#### Cu–As–Sb co-contamination

According to the study by De Gregori et al.^171^, the contaminated soils from Puchuncaví and Catemu valleys have Cu, As, and Sb concentration exceeding the reported critical values for this matrix, while at Catemu Valley, only Cu concentration was higher than this value. Moreover, at Puchuncaví valley, an apparent decrease in the three elements concentrations in soils as a function of the distance from the Ventanas industrial complex reflects the impact produced by Copper smelting in both valleys. While Cu–Sb correlation is significant for all the analyzed soil samples of this study, samples from Puchuncaví and Catemu valleys show more significant Cu–As, Cu–Sb, and Sb–As correlations. Based on this, these authors confirmed that high Cu, As, and Sb concentrations in these soils are coming from the Copper smelter, and the coal-fired thermoelectric power plant.

#### Cu, As, Cd, Pb, Zn, and Ti co-contamination

Ginocchio et al.^175^ and Sánchez de la Campa et al.^176^ also reported the Copper smelter at VIC as a source of significant amounts of Cu as well as As, Cd, Pb, Zn, and Titanium (Ti) to the atmosphere in the Puchuncaví Valley. Salmanighabeshi et al.^177^ arrived at the same conclusion based on a long term (2007–2011) soil monitoring campaign conducted around the industrial area of Puchuncaví-Ventanas involving the measurement of elemental concentration profiles, principal component analysis (PCA) and hierarchical cluster analysis (HCA), and application of several quantitative risk assessment indexes (geoaccumulation index, enrichment factor, contamination factor, contamination degree and integrated pollution index). Based on the distribution of metals in various particle size fractions of the soil from four sites of the Puchuncaví Valley, Parra et al.^178^ confirmed the emission of As and Cu enriched particulate matter from the Copper smelter. They noticed high Ca concentration in the finer fractions of the contaminated soils containing tenorite and Ca oxide, used to reduce Sulfer dioxide (SO_2_)emissions from the roasting process of Cu sulfide during the smelting activities. Poblete et al.^179^ performed spatial interpolation with the data obtained from 466 samples collected through stratified systematic sampling. Using the geostatistical kriging method, it showed concentrations of about 30 mg/kg in VIC vicinity. Moreover, As enrichment and its correlations with other elements helped these authors conclude that abnormal concentrations in both Puchuncaví and Quintero communes can be associated with Ventanas Copper smelter.

#### Remediation of Cu and As co-contaminated soil

Neaman et al.^180^ on the other hand studied the effectiveness of lime and compost for in situ immobilization of trace elements in the Cu and As contaminated acidic soils of the Puchuncaví Valley exposed to atmospheric depositions from a Copper smelter by using earthworms as bioindicators of toxicity. They found lime and compost treatments effective in significantly increasing soil pH and decreasing the soluble and exchangeable Zn, exchangeable Cu, and free Cu^2+^ activity. However, this compost treatment increased soluble Cu and soluble and exchangeable As. Also, the lime application did not affect earthworm reproduction, but compost increased cocoon and juvenile production. There was a spatial variability of soil properties within treatments in the field plots. They concluded that soil organic matter (SOM) was a positive factor for both cocoon and juvenile production, i.e., more SOM increased cocoon or juvenile production. The toxicity (negative) factor was total soil As, while total Cu and total As were well correlated (R^2^ = 0.80, p < 0.001), so it is pretty likely that some of the trends could have been masked. They recommended that the Chilean legislation on threshold concentrations of trace elements in soils should consider SOM content due to its effect on trace element solubility and bioavailability.

#### Health-risk assessment

Salmani-Ghabeshi et al.^181^ studied Punchuncaví Valley as a model environment for evaluating the spatial gradient of human health risk, mainly caused by trace elemental pollutants in the soil because of a range of anthropogenic emissions from VIC. These authors used soil elemental profiles in 121 samples from five selected locations representing different degrees of impact from the industrial source for human risk estimation. They found the distance to source dependent cumulative non-carcinogenic hazard indexes above 1 for children (1.5–4.4), ingestion being the most relevant risk pathway. They further confirmed the significance of health risk differences within the study area by statistical analysis, namely, anlaysis of variance (ANOVA) and hierarchical cluster analysis (HCA) of individual hazard index values at the five sampling locations. They found As to be the dominant factor causing unacceptable carcinogenic risk levels for children (< 10^−4^) at the two sampling locations closer to the industrial complex, whereas the risk was just in the tolerable range (10^−6^–10^−4^) for children and adults in the rest of the sampling locations at the valley.

#### As and other contaminants distribution map

Tapia-Gatica et al.^182^ evaluated potential human health and ecological risks associated with the soils in Puchuncaví and Quintero's townships contaminated by atmospheric deposition of sulfur dioxide and trace elements from the nearby VIC. They determined the spatial distribution of total As, Cu, Pb, and Zn concentrations in soil based on 245 topsoil samples used to generate continuous distribution maps. The background As, Pb, Cu, and Zn concentrations of the soils were 16, 35, 100, and 122 mg/kg, respectively, with 32, 77, and 35% of the study area showing As, Cu, and Pb concentrations, respectively, above the background level. Moreover, the As, Cu, and Pb concentrations were positively correlated, suggesting that their source to be the Ventanas Copper smelter. The carcinogenic risk due to As exposure was above the threshold value of 10^−4^ for children (1–5 years old) in 27% of the study area.

#### Leaves as biomonitor for As

Gorena et al.^183^ assessed Cupressus macrocarpa's usefulness as a biomonitor by studying leaf samples from five selected sites in the Punchuncaví Valley, located between 0.8 and 15 km away from VIC. They found high Cu (93.4–369 mg/kg) and As (7.6–12.7 mg/kg) values near the industrial complex exceed the phytotoxic levels reported in these plants with enrichment factor (EF) > 3000% for Cu and > 1300% for As. Through PCA and HCA, they identified six factors related to the industrial complex, traffic, and geogenic sources, which provided the most significant variance to the component connected to industrial activity, namely Copper smelter, and refinery.

#### Rainwater for As monitoring

Cereceda-Balic et al.^184^ studied the chemical composition of rainwater as an environmental pollution factor in the surroundings of VIC with the main objectives of assessing acidification and neutralization factors, measuring elemental pollutant levels, including calculation of enrichment factors and pollution sources assignment, and assessing the risk derived from elemental pollutant loads in rainwater, both for human use and natural ecosystems. Based on the analysis of 47 weekly rainwater samples collected during the winter (May–August) 2010 (24 samples) and 2011 (23 samples) at three sampling location with different degree of impact from the main emission sources, they found the elements emitted by metallurgical activities having significant enrichment values in the rainwater of the studied area through principal component analysis to identify the potential sources. Their risk assessment further showed that As content in rainwater is above the Chilean norms^185^ and the World health Organization^38–40^ guideline value (10 µg/L) for drinking water at some points in the study area around VIC.

Tapia Fernández et al.^186^ reported 131.2 ± 10.4 ppm of As in the soil in the ChiuChiu village of the Atacama Desert in northern Chile, and attributed it to the presence of volcanoes and geothermal activity by comparing As levels and the growth parameters among plants of the same genus. On the other hand, a study on public playgrounds for children assessed the soil quality in the Biobío Region of south-central Chile in 2018 identified dry weight average As concentrations varying from 18.82 to 23.53 mg/kg at four urban centers (Concepción, Talcahuano, Los Ángeles, and Tomé). However, based on the international guidelines for soil contamination^187–189^, Rodríguez-Oroz et al.^190^ concluded that there was no health risk for children in the studied playgrounds of the region.

## Controlling factors for As pathways

### Crustal thickness

The As-enrichment in the geothermal fields in CVZ (see Fig. 4 inset map that also shows the varying depth in kilometers to the Benioff zone) might also be related to the crustal thickness, which is over 70 km^112^, facilitating conditions for geothermal fluids to be enriched in other elements as well. Maity et al.^191^ underscored the difference in the As concentration in the geothermal fluids at the eastern and western margins of the Pacific Ring of Fire; i.e., up to 75 mg/L As concentrations in the hydrothermal discharges at the western margin (e.g., Los Humeros in Mexico, El Tatio in Chile, Copper River and Yellowstone National Park in the USA) and up to 6.2 mg/L at its eastern margin (e.g., Wairakei in New Zealand, Waiotapu, Ohaaki and Broadlands in New Zealand, Mt. Apo in the Philippines, Beitou in Taiwan)^192^. This difference can be explained in term of crustal thickness in Chile (e.g., El Tatio Geysers have reported As concentrations up to 45 mg/L; see "El Tatio geothermal field"), where there exists a positive correlation between the As concentration in thermal waters and crustal thickness^29^.

Tassara and Echaurren^31^ attributed the variations in the morphology and thickness of crustal roots underneath the high Andean cordilleras (~ 75 km below the Altiplano region; < 40 km along the southern Andes) and their spatial relation with surface topography and amounts of crustal shortening to large and small-scale variations in the isostatic mechanisms compensating the mountain chain and the processes that lead to the Andean orogeny. While associating the main compensation mechanism to thick and buoyant crustal roots in agreement with previous studies^193–195^, Tassara and Echaurren^31^ also attributed mantle thermal roots, elastic flexure, lateral density variations, and dynamic support from the mantle and lower crustal flow for partial compensation of the cordilleran topography. These favorable conditions for the uptake of As and other volatile elements by hydrothermal fluids to transport them to near-surface environments make a strong case for the crustal thickness an important controlling factor for As distribution in Chile. Aguilera et al.^196^ corroborated this idea by proposing that As could be related to a deep magmatic source characterized by a high content of volatile elements such as As in their work on hydrothermal alteration, fumarolic deposits, and fluids from Lastarria Volcanic Complex in northern Chile. Similarly, Guo et al.^197^ explained mantle-derived magmas' upwelling and associated high As concentrations at the surface in terms of crustal contamination and the partial melting of rocks in the very thick crust in Tibet. Tapia et al.^12^ reported a general positive correlation between crustal thickness and As concentrations in surface water and fluvial sediments, which tends to decrease progressively from northern to southern Chile. These authors attributed this trend to decreased shortening of the Andes in southern Chile, as related lower crustal thickness induces lower crustal contamination, acting as an As-poor provenance for the sediments and surface water.

### Geological structure

Apart from the aforementioned solid and liquid phase As inputs (see "Principal geogenic As sources"), attributed to the lithology and geothermal and volcanic fluids, respectively, another important control for As enrichment of aquifers in a basin is its structural setting. For example, in Duero Basin, Spain, Giménez-Forcada and Smedley^198^ accounted basin morphology, prolongation of faults, especially division of the underlying basement into blocks, producing discrete hydrodynamic environments, for the spatial variations in hydrochemical composition, including As speciation. In this basin, these authors found the groundwater extracted from the deep boreholes along the main fault lines oxidizing, alkaline, Sodium-Bicarbonate (Na-HCO_3_) type with relatively high As concentrations in pentavalent [As(V)] form, up to 241 μg/L. Although some workers have studied structural control for geochemical variation thermal waters and recharge of geothermal waters in Chile (see "Geothermal activities"), it is yet to be done for groundwater chemistry, particularly As concentration and speciation^199,200^.

Hydraulic connections between the basins could lead to the contamination of a basin without local contaminants. For example, the heavy precipitation of March 2015 that led to flooding in the Atacama Region mobilized dissolved, and particulate As in the surface environment and created a hydraulic connection between the El Salado Alto and El Salado Bajo basins. As a result, the average concentration of dissolved As increased from 2 µg/L in 2014 to 287 µg/L 2 months after the flooding, with a subsequent decrease to 19 µg/L one year later, following the restoration of the hydraulic disconnection between El Salado Alto and El Salado Bajo^13^.

Another structural control could be the hydraulic gradient, the lower one providing more residence time for groundwater^201,202^. Smedley and Kinniburgh^203^ pointed out that whether released As remains at problematic levels in groundwater depends not only on whether there are biogeochemical reactions that retard As transport but also upon the hydrologic and hydrogeologic properties of the aquifer, such as flow velocity and dispersion. If the kinetics of As release is slow, and groundwater residence time is short, then As concentrations may not increase to the point where groundwater would be considered contaminated. Conversely, reactions that mobilize As are rapid and residence time is extended, then As can accumulate in groundwater such that concentrations become hazardous, e.g., West Bengal (India), Bangladesh^204–206^. To assess hydrogeological control for As concentration in Chile, more in-depth studies are required in the basins where such controls are expected to play a role in As concentration and speciation.

### Evapoconcentration and salinity

Tapia et al.^10,11^ attributed relatively low As in southern Chile to increased dilution due to more precipitation and the absence of As-rich evaporites (e.g., Altiplano-Puna plateau^17^), saline precipitates^13,14^, brines (TDS > 50,000 mg/L, average As 14,200 mg/L^13,14,207^), and saline water (As up to 87,600 µg/L^207,208^) present in dry northern Chile. Moreover, Tapia et al.^10,11^ concluded that the high concentrations of As well above the upper continental crust average do not reflect the geochemistry of volcanic or plutonic rocks of the region. Elevated As concentrations in the Atacama Desert's closed basins can be attributed to high evaporation rates due to low precipitation and hyperarid climate since Miocene.

In terms of salinity, the highest concentrations of As are found in brines (up to 87,600 µg/L^207,208^), followed by saline water and hot springs in the CVZ (up to 25,000 and 45,000 µg/L, respectively^24^), saline water in PFS (up to 10,400 µg/L^25^), and hot springs in the SVZ (Pemehue, up to 10,000 µg/L^132^). In brines (TDS ≥ 50,000 mg/L), dissolved As in the Chilean Altiplano-Puna plateau presents an average of 14,200 µg/L^13,14,207^.

Although high salinity in northern Chile can be attributed to evapoconcentration^208^ associated with high As concentration^29^, the same is not valid for the reported high salinity in southern Chile's thermal waters. Consequently, the evaporation and salinity as controlling factors for As should be treated separately.

### Elevation

The rapid exhumation of the Andean Arc and resulting erosion leaves the active hydrothermal system capless in many segments^209^, leading to a pervasive nature (widespread) of the hydrothermal fluids and resulting alteration, rather than being localized, fault controlled. They are the primary As sources in the elevated areas. This control can be observed in terms of the variation of As concentration in some rivers as a function of elevation in Chile. For example, water quality monitoring throughout the Copiapó River and its tributaries show As concentrations below the Chilean guideline for drinking water of 10 µg/L^185^ in most of the river sections, except for the headwaters section and a downstream sector (Piedra Colgada), where the river is fed by highly mature As-enriched groundwater^174^. Similarly, Vega et al.^210^ reported relatively higher As concentrations in the streams and groundwater in the Loa, Elqui, and Lluta river catchments. This could be attributed to the presence of the mineralized zones in the headwaters area, as in Copiapó River's case, geothermal discharges, as in Loa River, fed by its major As contributor Salado River, originating from ETGF, or dissolution of volcanic glass present in volcanic rocks and ashes. This association of As with elevation needs further investigation.

### Other potential sources and controlling factors

Coal deposits and hydrocarbon reservoirs are other two well-known geogenic sources of As across the world (viz. China, Japan, Turkey), including Latin America (viz. Brazil); however, there is no information currently available on potential As pollution related with either of these energy resources in the country. Although there are studies on As contamination originated from coal-fired (thermoelectric) power plants and petroleum refineries^211^, there are scarce reports on coal and hydrocarbon deposits as potential As sources^212^.

Also, the role of agricultural activity in accelerating As contamination needs to be investigated, as a significant amount of As could be released from the sediments due to the agriculture activity and contaminate shallow groundwater resources, as in Bengal delta, India^213^. Work on As translocation in rice cultivation and its implication for human health by Bastías and Baldarrian^214 ^and recent work of Moncada et al.^215^ dealing with the remediation of agricultural soils with long-term As and Cu contamination are efforts towards filling this gap.

## Conclusions

Arc magmatism and associated geothermal activities are the principal primary As sources, present across the Chilean Andes, except for the Pampean Flat Slab and Patagonian Volcanic Gap. Arsenic from the Andean Cordillera is transported to the lowlands on its either side through exogenic forces like water and wind, leading to As concentration in the sediments deposited in the intermediate basin between the Andes and the Coastal Ranges in Chile towards west or eastward to the Andean foreland basin on the Argentine side. These As-rich sediments are formed either through accelerated erosion of the magmatic arc rocks (primary geogenic source) due to the rapid exhumation of the Andes, or As-rich vitric rhyolitic proportion of the volcanic ash. Arsenic transportation is also possible in solution following the dissolution of volcanic glass locally within the highlands or during the transport of ash by rivers. Geothermal waters rich in As (up to 4500 mg/L) contaminate both groundwater and surface water; viz. Salado River originating from El Tatio Geysers contribute As to the Loa River, the longest one in Chile that drains a major part of the Atacama Desert. The metallogenic belts extending from the country’s far north to the south-central part, and also stretching far south, are the second most important geogenic As source. Natural leaching of As from these mineralized zones contaminates the groundwater and soil horizon in their contact. Associated mining and metallurgical activities further accelerate this process, e.g., air and soil contamination from the smelter emissions. Some of the affected areas in various parts of the country are categorized as “sacrifice” o “saturated” zones. Moreover, a positive correlation between As concentration and crustal thickness, elevation, and evaporation rate envisages them as controlling factors for As distribution, which requires further investigation to understand the mechanism of As dissemination controlled by these factors.

## Data Availability

The datasets analyzed during the current study are summarized in this article and are available from the corresponding author on reasonable request. Additionally, the used data can be accessed from the cited references.
